# Candidate Biomarkers for Crohn’s Disease: Hub Genes and Regulatory miRNAs Identified by Bioinformatics Analysis

**DOI:** 10.1155/bri/4628067

**Published:** 2026-02-05

**Authors:** Han Wang, Mengdie Shen

**Affiliations:** ^1^ Department of Gynecology and Obstetrics, Women’s Hospital, School of Medicine, Zhejiang University, Hangzhou, 310006, Zhejiang, China, zju.edu.cn; ^2^ Department of Internal Medicine, Women’s Hospital, School of Medicine, Zhejiang University, Hangzhou, 310006, Zhejiang, China, zju.edu.cn

**Keywords:** bioinformatics analysis, Crohn’s disease, differentially expressed genes, hub genes, microRNAs

## Abstract

**Background:**

Crohn’s disease (CD) is a chronic, complex inflammatory condition that can affect the entire digestive tract, most commonly the terminal ileum. The exact cause of CD remains unknown. Bioinformatics was used in this study to identify the differentially expressed genes (DEGs) and microRNAs (miRNAs) that show potential as diagnostic and therapeutic agents in treating CD.

**Materials and Methods:**

Datasets were downloaded from the Gene Expression Omnibus database and filtered. DEGs between CD samples and healthy control samples were identified using the GEO2R tool (including GEOquery and Linear Models for Microarray Analysis), and Kyoto Encyclopedia of Genes and Genomes/Gene Ontology enrichment analyses were conducted as part of the study to gain deeper insights into the data. A network depicting protein–protein interactions was established and visualized using the STRING database and Cytoscape software, and hub genes were identified and extracted utilizing the cytoHubba program. Cytoscape and miRTarBase were used to construct the miRNA–hub gene regulatory network and predict the potential miRNAs associated with the DEGs. The hub genes were analyzed further using ROC curves and combined ROC curve analyses of the GSE179285, GSE186582, and GSE112366 datasets. A regularized LASSO regression model was constructed to reduce the risk of overfitting.

**Results:**

Three datasets (GSE179285, GSE186582, and GSE112366) were selected. A comprehensive analysis of the three datasets revealed 60 DEGs that showed significantly altered expression levels, including 44 upregulated genes and 16 downregulated genes. Ten different algorithms were randomly used, and three hub genes (*CXCL1*, *CXCL2*, and *CXCR2*) were identified. Based on the miRNA–hub gene regulatory network, hsa‐miR‐1‐3p and hsa‐miR‐335‐5p were recognized as potentially vital miRNAs.

**Conclusion:**

Three hub genes (*CXCL1*, *CXCL2*, and *CXCR2*) and two miRNAs (hsa‐miR‐1‐3p and hsa‐miR‐335‐5p) are postulated to play a role in the initiation and progression of CD, thereby offering potential as biomarkers for this condition.

## 1. Introduction

Crohn’s disease (CD) is a chronic, complex inflammatory condition that can affect the entirety of the digestive tract, but most commonly the ileum. In the past few decades, the incidence of inflammatory bowel disease (IBD) including CD and ulcerative colitis has increased worldwide [1, 2]. The exact etiology of CD remains unknown. It is currently widely accepted that the pathogenesis of CD is closely associated with genetic susceptibility, environmental factors, and immune imbalance [3, 4]. To date, there is a lack of strict and recognized criteria to diagnose CD. The clinical manifestations of CD are diverse, among which abdominal pain, diarrhea, fever, and weight loss are the most common manifestations. Moreover, extraintestinal manifestations and systemic symptoms can occur, which can seriously affect the physical and mental well‐being of patients and increase the burden on social medical resources [5].

Endoscopic mucosal healing has become a major treatment target and endpoint in recent years owing to its clinical and biological efficacy. Numerous researchers are targeting higher therapeutic goals such as transmural healing and histological remission [6]. Since the advent of biological agents, the clinical outcomes of CD have improved significantly, with a substantial increase in clinical remission rates [7]. However, some patients are not responsive to biological agents. Therefore, it is crucial to identify the specific details of the molecular mechanisms of CD to develop personalized treatment strategies.

## 2. Materials and Methods

### 2.1. Microarray Data

Gene Expression Omnibus (GEO) database (https://www.ncbi.nlm.nih.gov/geo/) [8] is a gene‐expression database established and maintained by the National Center for Biotechnology Information in the United States. It contains high‐throughput gene‐expression data contributed by various research institutions all over the world. From the GEO database, we downloaded the microarray data of inflamed ileal mucosal tissue of patients with CD that met the criteria as well as healthy control ileal mucosal tissue data. We finally selected GPL6480 platform dataset GSE179285 [9], GPL570 platform dataset GSE186582 [10], and GPL13158 platform dataset GSE11236 [11]. The GSE179285 dataset included 33 CD samples and eight healthy control samples; the GSE186582 dataset included 196 CD samples and 25 healthy control samples; and the GSE112366 dataset included 141 CD samples and 26 healthy control samples.

### 2.2. Identification of Differentially Expressed Genes (DEGs)

DEGs were identified between samples of inflamed ileum from CD patients and healthy control ileum samples using the GEO2R online tool [12, 13]. GEO2R utilizes GEOquery and Linear Models for Microarray Analysis (Limma) for conducting differential expression analysis, using the originally processed data tables provided by the submitter as the input for DEG identification. GEOquery processes GEO data into R data structures that are compatible with other R packages. Limma is a statistical tool designed to identify DEGs in microarray data. It accommodates various experimental designs and data formats and incorporates multiple testing corrections on *p* values to mitigate the risk of false positives, thereby enhancing the reliability of the differential expression analysis. Statistical significance was defined as |Log fold change (FC)| > 1 and adj. *p* value < 0.05.

### 2.3. Gene Ontology (GO) Functional Annotation and Kyoto Encyclopedia of Genes and Genomes (KEGG) Analysis for DEGs

ClusterProfiler [4.4.4] in the R package was used for KEGG and GO enrichment analyses for core targets. The significance level was set at *p* < 0.05, and a false discovery rate threshold of < 0.05 was set as the cutoff criterion to determine statistical significance. GO [14] is a standardized classification system consisting of a hierarchical structure with three levels, including biological processes (BPs), molecular functions, and cellular components. KEGG [15, 16] is a comprehensive database that provides a platform for understanding biological systems at the molecular level. It is widely used for pathway analysis, network visualization, and interpretation of high‐throughput data in genomics, bioinformatics, and system biology research.

### 2.4. Construction of a Protein–Protein Interaction (PPI) Network and the Identification of Core Genes

Using the STRING database (https://string-db.org/), a PPI network was constructed. The results were imported into Cytoscape software (Version 3.7.2), and key nodes were selected for the visualization of the molecular interaction network. Based on the PPI network, the hub genes were identified using the cytoHubba plugin.

### 2.5. Hub Gene–Related miRNAs

MiRTarBase [17] is the experimentally validated microRNA (miRNA) target interaction database that provides the most updated collection by comparing it with other similar, previously developed databases. MiRTarBase 9.0 is supported by experimental evidence (reporter assay, western blotting, qPCR, microarray, and CLIP‐seq). We mapped the hub genes to their respective miRNAs using miRTarBase. Lastly, network analysis and visualization of these hub genes and miRNAs were performed using Cytoscape 3.7.2.

### 2.6. Verification of Hub Genes in CD and Construction of the Least Absolute Shrinkage and Selection Operator (LASSO) Model

ROC curves and combined ROC curve analyses were performed to evaluate the diagnostic performance of the identified hub genes. The area under the curve (AUC) values, a key metric for assessing performance in the ROC curve, were calculated using the “pROC” package (Version 1.18.0) in R software (Version 4.2.1). Notably, “pROC” automatically adjusts the data order by default to ensure the accuracy of ROC curve generation and AUC calculation. All ROC curve plots and associated visualizations were undertaken using the “ggplot2” package (Version 3.4.4) in R, with the application of consistent formatting to enhance readability and adherence to academic publishing standards. Potential biomarkers for the diagnosis of CD were evaluated using a LASSO) model implemented in the “glmnet” package in R for the analysis of the preprocessed dataset. Key outputs, including the regularization parameter (*λ*), likelihood values, and misclassification rates were derived, and the data were visualized to assist the interpretation of the results.

## 3. Results

### 3.1. Identification of DEGs

Three datasets (GSE179285, GSE186582, and GSE112366) were selected from the GEO database, and GEO2R was employed to analyze the DEGs between healthy control samples and inflamed ileum mucosal samples from patients with CD. Details of the three datasets are shown in Table 1. A comparative analysis between CD samples and healthy control samples revealed a total of 1171 DEGs, of which 660 were upregulated and 511 were downregulated. Among them, the GSE179285 dataset contained 239 upregulated genes and 141 downregulated genes; the GSE186582 dataset contained 324 upregulated genes and 327 downregulated genes; and the GSE112366 dataset included 97 upregulated genes and 43 downregulated genes. DEGs were identified through a comparative analysis of the gene expression profiles between the healthy controls and the samples from patients with CD. Figure 1 depicts the profiles of gene expression for DEGs across the three datasets. Following additional screening, these genes were identified, and Venn diagrams were used for illustration. The analysis revealed 60 DEGs that exhibited significant differential expression across the three groups (Figure 2), with 44 upregulated genes and 16 downregulated genes.

**TABLE 1:** Details of the selected datasets.

Characteristics	GSE179285	GSE186582	GSE112366
Reference	Keir et al. [9]	Ngollo et al. [10]	VanDussen et al. [11]
Platform	GPL6480	GPL570	GPL13158
Healthy controls (*n* = 59)	8	25	26
Crohn’s disease (*n* = 370)	33	196	141

FIGURE 1Volcano plots illustrating the differentially expressed genes (DEGs) between the healthy control samples and CD samples. DEGs from the GSE179285, GSE186582, and GSE112366 datasets are displayed individually. Upregulated genes are denoted by red points and downregulated genes by blue points; genes with no significant differences are shown in gray.

**Figure:**
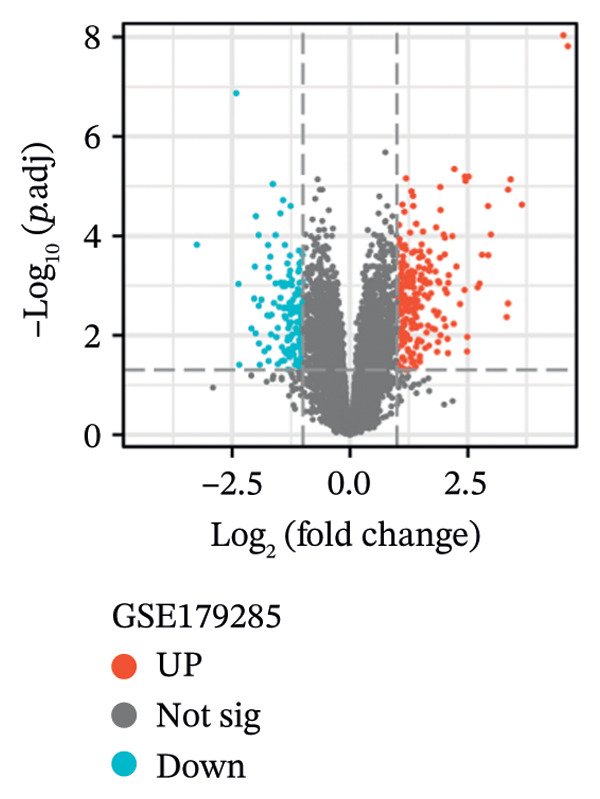
(a)

**Figure:**
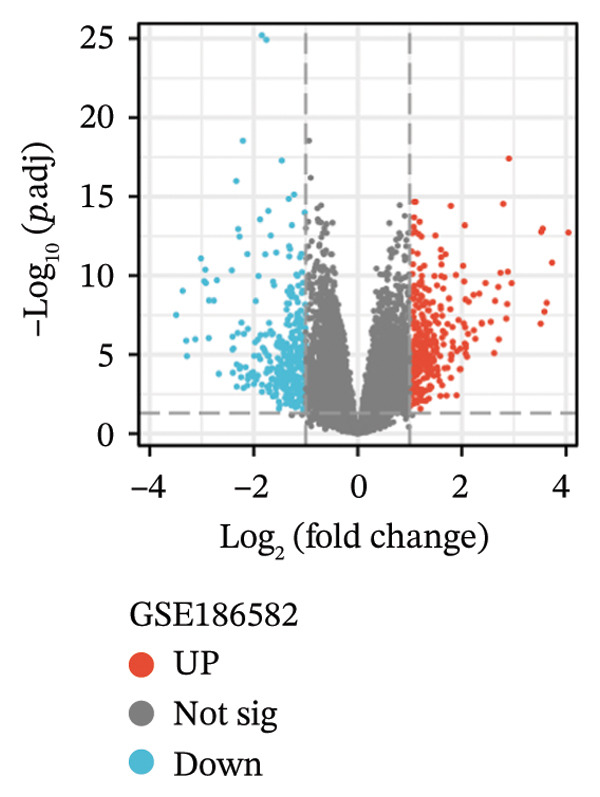
(b)

**Figure:**
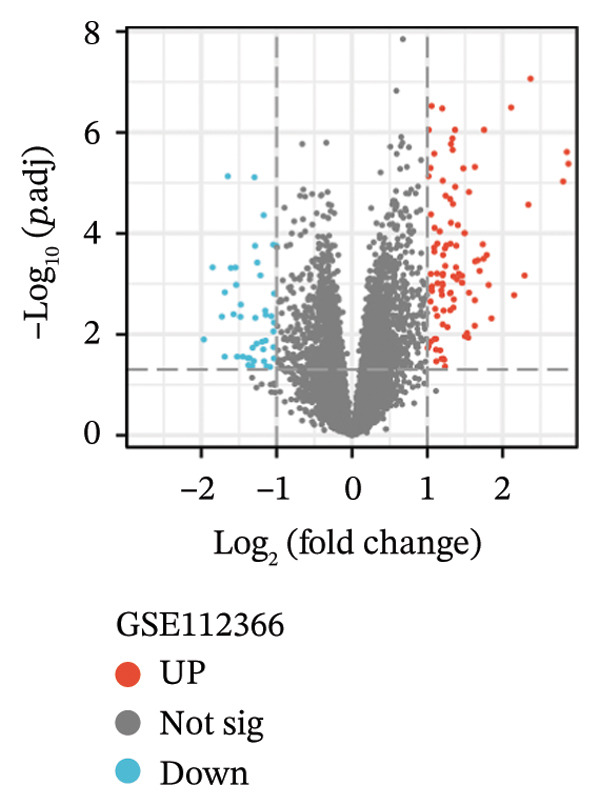
(c)

FIGURE 2Venn diagrams illustrating the overlap of differentially expressed genes (DEGs) among the four datasets obtained from the Gene Expression Omnibus (GEO) database. Panels (a) and (b) demonstrate the intersection of upregulated and downregulated genes across the GSE179285, GSE186582, and GSE112366 datasets, respectively.

**Figure:**
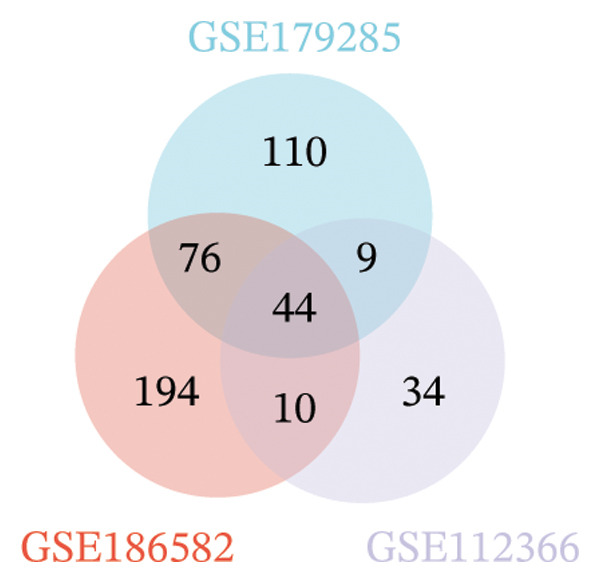
(a)

**Figure:**
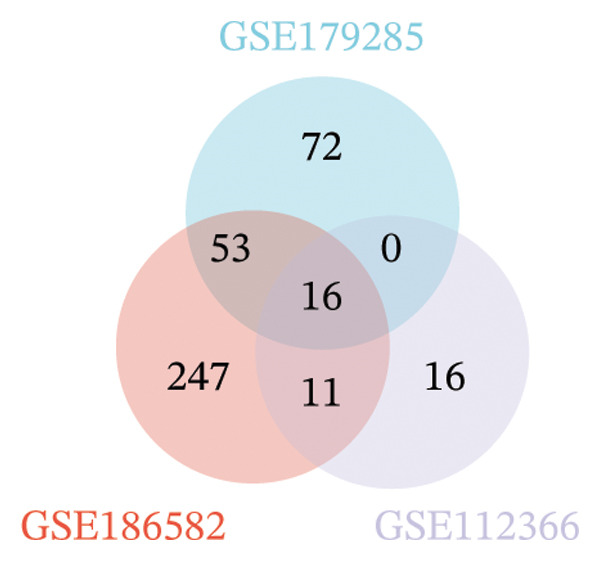
(b)

### 3.2. KEGG and GO Enrichment Analyses of DEGs

Separate functional enrichment analyses of upregulated and downregulated genes were conducted to determine the biological functions of DEGs. The results from GO analysis indicated that the upregulated genes were primarily enriched in categories such as collagen‐containing extracellular matrix, leukocyte migration, endoplasmic reticulum lumen, and extracellular matrix structural constituent (Figure 3(a)). Conversely, the downregulated genes exhibited significant enrichment in the apical part of the cell, apical plasma membrane, brush border, and olefinic compound metabolic process (Figure 3(b)). KEGG pathway analysis revealed significant enrichment among DEGs in cytokine–cytokine receptor interaction, viral protein interaction with cytokines and the cytokine receptor, and protein digestion and absorption (Figure 3(c)).

FIGURE 3GO and KEGG pathway enrichment analyses to identify DEGs associated with CD. (a) Bubble plot illustrating enriched GO terms with upregulated DEGs. (b) Bubble plot illustrating enriched GO terms with downregulated DEGs. (c) Enrichment analysis of KEGG pathways for the DEGs associated with CD.

**Figure:**
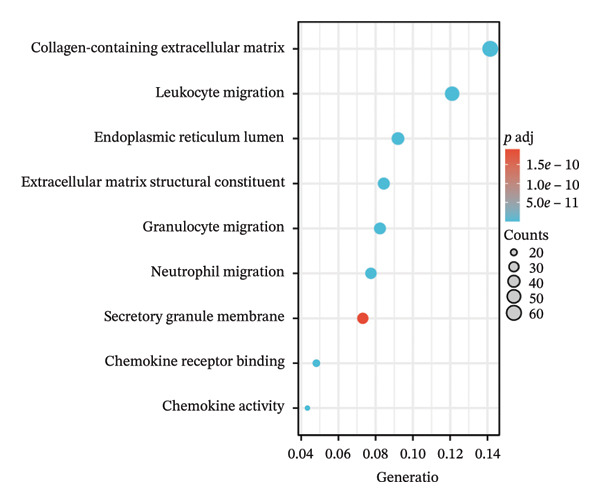
(a)

**Figure:**
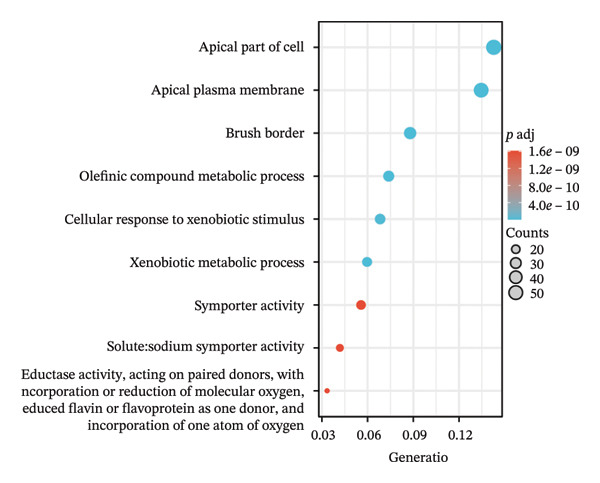
(b)

**Figure:**
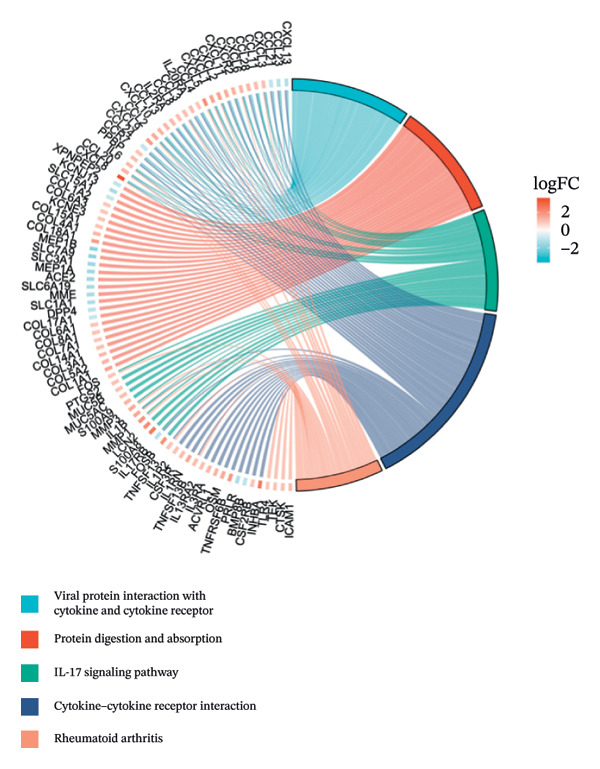
(c)

### 3.3. Construction of PPI Networks, Modular Analysis, and Identification of Hub Genes

PPI analysis of DEGs was performed using the STRING database, and the outcomes were subsequently visualized using the Cytoscape platform (Figure 4(a)). The MCODE plugin in Cytoscape was applied to screen gene cluster modules with a score of > 5, resulting in Cluster 1 (score 7.0, including 11 nodes and 35 edges) and Cluster 2 (score 6.8, including 11 nodes and 34 edges) in the PPI network (Figures 4(b), 4(c)). The cytoHubba plugin in Cytoscape was employed to assign scores to each node gene, employing ten different algorithms randomly, including MCC, DMNC, MNC, EPC, Degree, BottleNeck, Closeness, Eccentricity, Radiality, and Betweenness. The R package “UpSet” was used to filter the top 20 genes from the results of each algorithm, which facilitated the identification of three hub genes, namely, *CXCL1*, *CXCL2*, and *CXCR2*, all of which were upregulated in patients with CD (Figures 4(d), 4(e), 4(f), 4(g)).

FIGURE 4PPI network and gene cluster modules. (a) Cytoscape was used to visualize the PPI network of DEGs. (b)–(c) Top two important gene cluster modules using the MCODE plugin. (d) Degree of interaction of the three hub genes. (e)–(g) Expression levels of the three hub genes in the GSE179285, GSE186582, and GSE112366 datasets, respectively.

**Figure:**
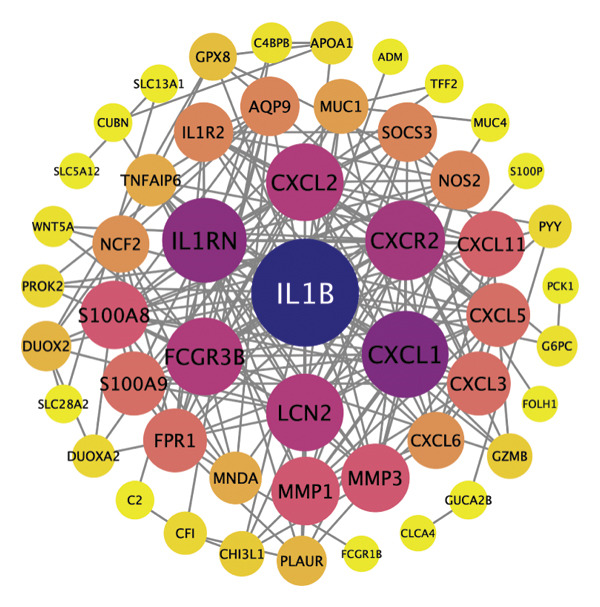
(a)

**Figure:**
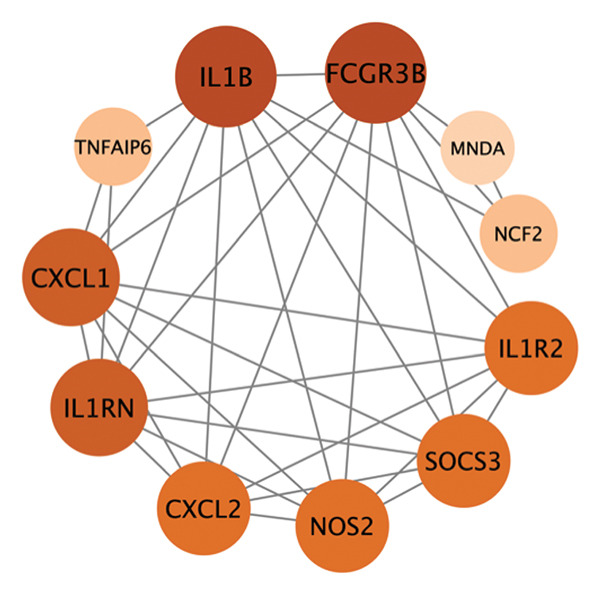
(b)

**Figure:**
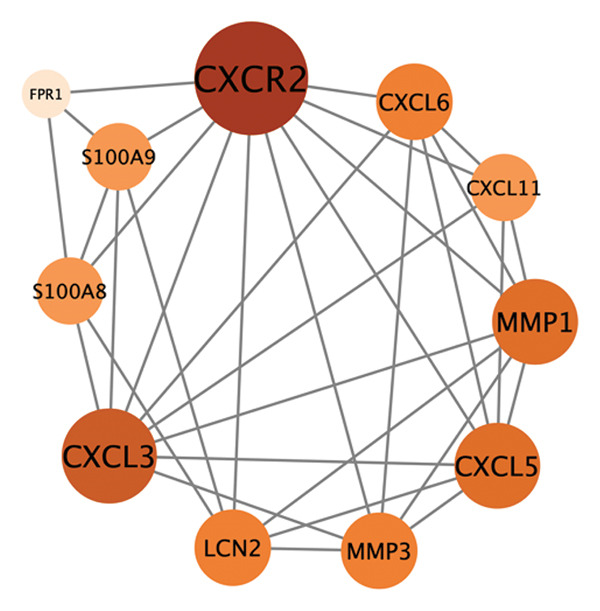
(c)

**Figure:**
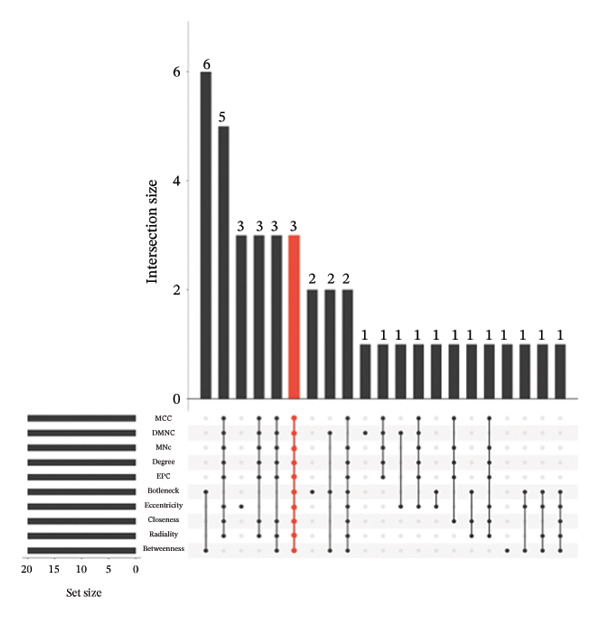
(d)

**Figure:**
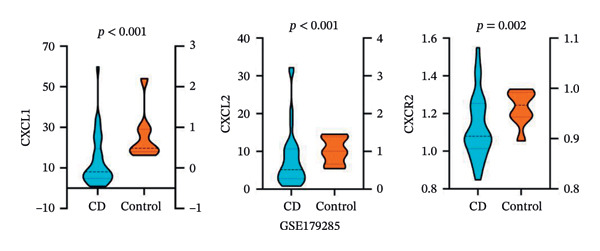
(e)

**Figure:**
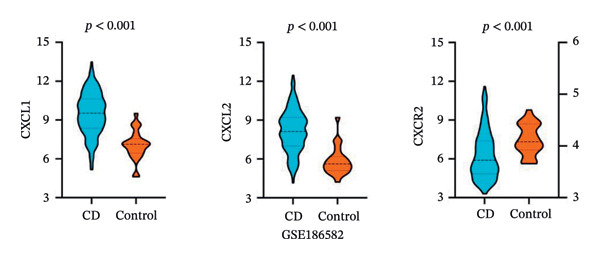
(f)

**Figure:**
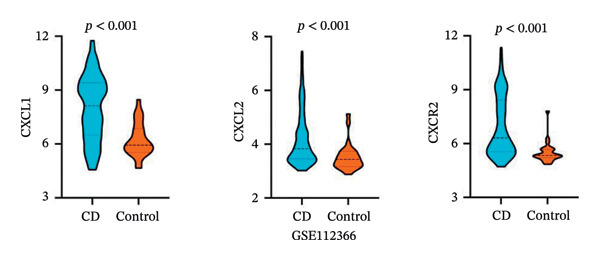
(g)

### 3.4. Analysis of Hub Genes

Table 2 presents the symbols, abbreviations, and individual functions of the three hub genes. Functional enrichment analysis identified that these three hub genes were predominantly associated with BPs, including chemokine‐mediated signaling pathway, response to chemokines, and cellular response to chemokines. Meanwhile, KEGG analysis primarily focused on cytokine–cytokine receptor interaction, chemokine signaling pathway, viral protein interaction with cytokines and cytokine receptor, and epithelial cell signaling in *Helicobacter pylori* infection (Figures 5(a), 5(b), Table 3).

**TABLE 2:** Three hub genes and their functions.

Gene symbol	Description	Function
*CXCL1*	C‐X‐C motif chemokine ligand 1	Growth‐regulated alpha protein; possesses chemotactic properties that attract neutrophils. Plays a pivotal role in inflammation and modulates endothelial cells via an autocrine mechanism
*CXCL2*	C‐X‐C motif chemokine ligand 2	Activated monocytes and neutrophils produce this substance, which is expressed at sites of inflammation
*CXCR2*	C‐X‐C Chemokine Receptor Type 2	Receptor for Interleukin (IL)‐8, a powerful neutrophil chemotactic factor. Binding of IL‐8 to the receptor activates neutrophils.

FIGURE 5Functional enrichment analysis of the three hub genes. (a) Bubble plot illustrating enriched GO terms with hub genes. (b) Enrichment analysis of KEGG pathways for hub genes.

**Figure:**
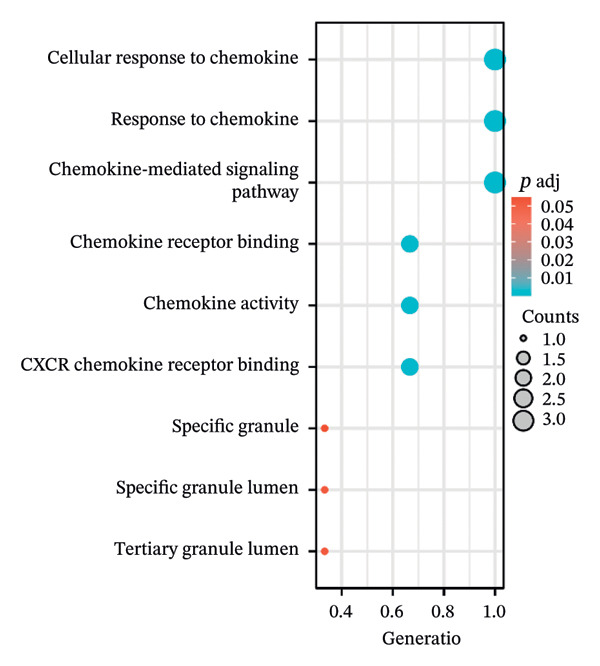
(a)

**Figure:**
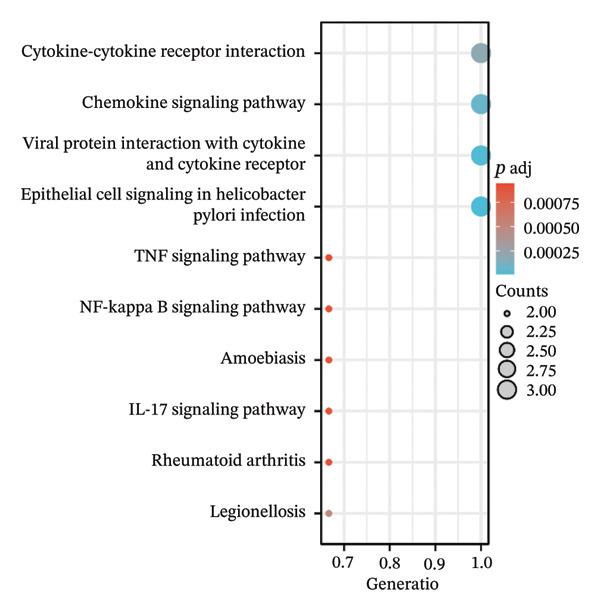
(b)

**TABLE 3:** Functional enrichment analysis of the 3 hub genes.

Term	Description	Count	*p* value	Gene symbol
GO: BP	Cellular response to chemokine	3	1.33156487405253*E* − 07	*CXCL1/CXCL2/CXCR2*
GO: BP	Response to chemokine	3	1.33156487405253*E* − 07	*CXCL1/CXCL2/CXCR2*
GO: BP	Chemokine‐mediated signaling pathway	3	1.02562285239354*E* − 07	*CXCL1/CXCL2/CXCR2*
KEGG	Cytokine–cytokine receptor interaction	3	4.67183594463347*E* − 05	*CXCL1/CXCL2/CXCR2*
KEGG	Chemokine signaling pathway	3	1.28096957269623*E* − 05	*CXCL1/CXCL2/CXCR2*
KEGG	Viral protein interaction with cytokines and cytokine receptor	3	1.78365923726389*E* − 06	*CXCL1/CXCL2/CXCR2*
KEGG	Epithelial cell signaling in *Helicobacter pylori* infection	3	6.03818841359465*E* − 07	*CXCL1/CXCL2/CXCR2*

### 3.5. Establishment of a Regulatory Network of miRNA–Hub Genes

**FIGURE 6:**
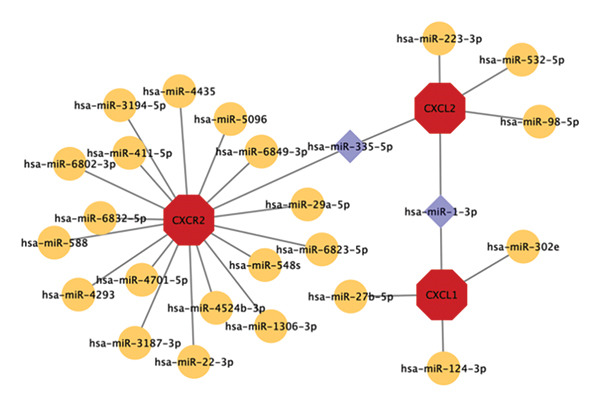
miRNA–mRNA regulatory network. Red octagons indicate the three hub genes. Purple diamonds indicate the miRNAs with strong connective properties to the hub genes.

Using miRTarBase and Cytoscape, the miRNA–mRNA regulatory network was built, which included 28 nodes (25 miRNAs, three mRNAs) and 27 edges (Figure 6). Has‐miR‐1‐3p and has‐miR‐335–5p had two target genes each (*CXCL2* and *CXCL1*; and *CXCL2* and *CXCR2*, respectively).

### 3.6. Verification of Hub Genes and Construction of the LASSO Model

**FIGURE 8:**
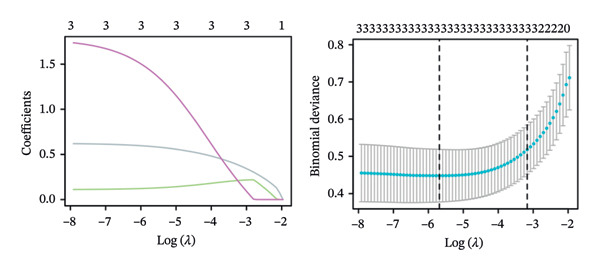
LASSO regularization. LASSO coefficient pathway plot and tenfold cross‐validation binomial deviance curve. Each curve represents the regularization coefficient of a gene. The vertical dashed lines indicate the optimal *λ* values.

To evaluate the predictive value of the three hub genes, the individual and combined ROC curves of these genes were analyzed using the GSE179285, GSE186582, and GSE112366 datasets. All three genes were found to have significant predictive performance following assessments in patients with CD and healthy controls. In the GSE179285 dataset, the AUC values were 0.970 for CXCL1 (95% CI = 0.925–1.000), 0.936 for CXCL2 (95% CI = 0.861–1.000), and 0.845 for CXCR2 (95% CI = 0.727–0.962). In the GSE186582 dataset, the AUC values were 0.890 for CXCL1 (95% CI = 0.836–0.944), 0.884 for CXCL2 (95% CI = 0.817–0.952), and 0.905 for CXCR2 (95% CI = 0.865–0.945). A combined analysis integrating these three genes yielded an overall AUC of 0.931 (95% CI = 0.897–0.964). The AUC values in the GSE112366 dataset were 0.784 for CXCL1 (95% CI = 0.711–0.858), 0.737 for CXCL2 (95% CI = 0.636–0.838), and 0.798 for CXCR2 (95% CI = 0.720–0.876). The combined analysis of the three genes in this dataset indicated an overall AUC of 0.812 (95% CI = 0.736–0.887). Among these genes, CXCL1 and CXCL2 in the GSE179285 dataset exhibited perfect discrimination between positive and negative samples. Specifically, the two genes showed complete separation, with no overlap in their distributions between the positive and negative groups, indicating their unsuitability for use as joint indicators. This observation may be attributed to two potential factors: first, the small sample size of the GSE179285 dataset, and second, strong interfeature correlations between CXCL1 and CXCL2. To address this limitation and avoid the overestimation of the predictive value of individual genes, we undertook a joint ROC analysis of all three datasets to provide a more comprehensive verification of the predictive performance of the hub genes. It is worth noting that the AUC values for each of the hub genes exceeded 0.7, indicating good predictive performance (Figures 7(a), 7(b), 7(c), Supporting data 1). The findings of the LASSO model indicated that the onset and progression of CD was linked to the expression of the genes, namely, CXCL1, CXCL2, and CXCR2 (Figure 8).

FIGURE 7ROC analysis of the three hub genes. (a) ROC curves of the three hub genes in the GSE179285 dataset. (b)–(c) ROC curves of the three hub genes and joint ROC curves of CXCL1, CXCL2, and CXCR2 in the GSE186582 and GSE112366 datasets.

**Figure:**
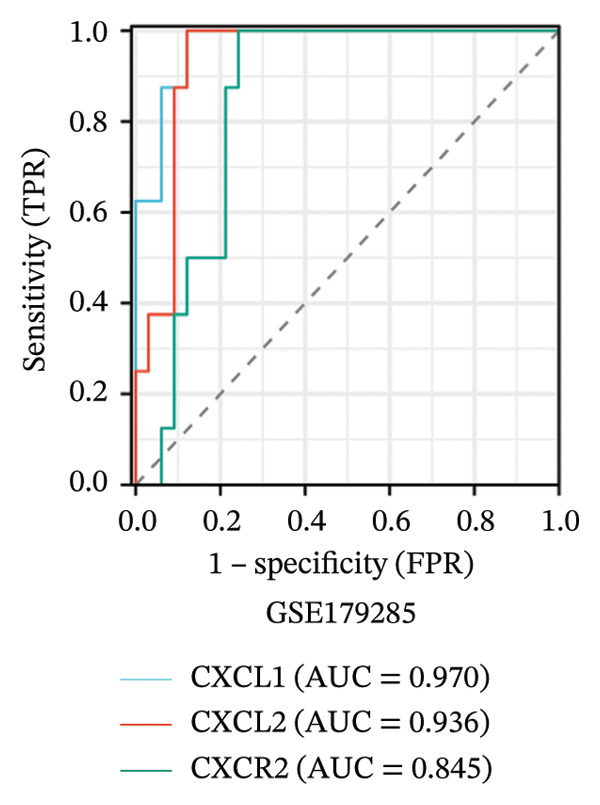
(a)

**Figure:**
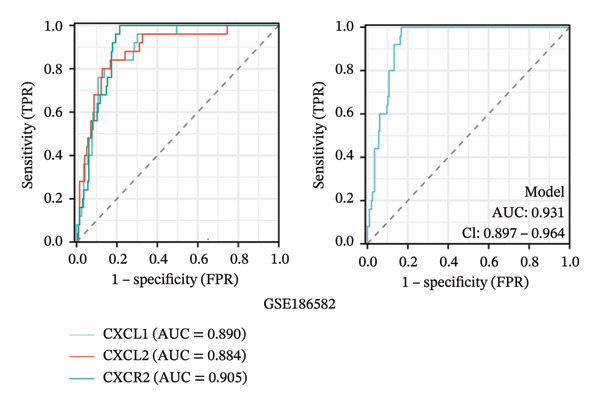
(b)

**Figure:**
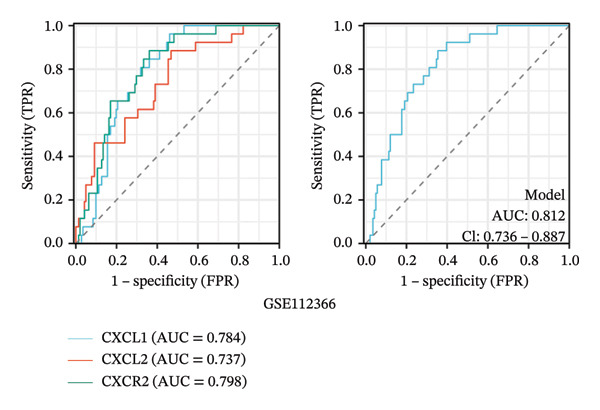
(c)

## 4. Discussion

CD is a complex, chronic intestinal inflammatory granulomatous disease with unknown etiology that can affect the entire digestive tract, but the terminal ileum and adjacent colon are more commonly affected. The disease persists and has a lifelong tendency to relapse, causing serious disturbances to patients’ lives [18]. In current clinical practice, while biological agents can target intestinal lesions precisely and significantly alleviate clinical symptoms, a subset of patients gradually develops resistance after treatment, leading to diminished therapeutic efficacy or even complete treatment failure [19, 20]. Therefore, there is an urgent need to explore novel diagnostic indicators and therapeutic targets to address this clinical dilemma. Against this backdrop, the present study employed bioinformatics approaches to analyze DEGs between the inflamed ileum samples from CD patients and healthy ileum tissues. The aim was to identify molecular markers for CD with potential for clinical translation, thereby providing a theoretical basis for the precise diagnosis and targeted therapy of the disease.

The expression of 60 DEGs was found to be significantly altered in the three datasets (GSE179285, GSE186582, and GSE112366), including 44 upregulated and 16 downregulated genes. The results of the GO analysis indicated that the upregulated genes were primarily enriched in the collagen‐containing extracellular matrix, leukocyte migration, endoplasmic reticulum lumen, and extracellular matrix structural constituents. These findings are strongly consistent with the pathological characteristics of CD, such as collagen deposition and inflammatory cell infiltration following intestinal mucosal injury. In patients with CD, intestinal mucosal tissues often exhibit abnormal remodeling of the extracellular matrix, resulting in thickening and fibrosis of the intestinal wall [21]. The downregulated genes were significantly enriched in the apical part of the cell, apical plasma membrane, brush border, and olefinic compound metabolic processes. This suggests impaired polarity of intestinal epithelial cells, accompanied by disrupted absorptive functions, in patients with CD, consistent with the common clinical manifestations such as malabsorption and weight loss [22]. KEGG pathway analysis indicated a notable enrichment of the DEGs in the cytokine–cytokine receptor interaction, viral protein interaction with cytokines and cytokine receptors, and protein digestion and absorption. Activation of cytokine–cytokine receptor interaction is closely associated with the initiation and amplification of intestinal inflammation in CD. In PPI networks, three of the 60 genes (*CXCL1*, *CXCL2,* and *CXCR2*) were found to interact highly. The results from GO analysis revealed a significant enrichment of the three hub genes in the chemokine‐mediated signaling pathway, response to chemokines, and cellular response to chemokines, whereas KEGG analysis primarily focused on the cytokine–cytokine receptor interaction, chemokine signaling pathway, viral protein interaction with cytokines and cytokine receptor, and epithelial cell signaling in *H. pylori* infection.

Zhang et al. [23] have documented that in the animal models of TNBS‐induced colitis, there was an increase in the serum levels of the pro‐inflammatory cytokines, including tumor necrosis factor (TNF)‐α, Interleukin (IL)‐1β, IL‐6, IL‐17A, and interferon‐γ, accompanied by a decrease in those of the anti‐inflammatory cytokine IL‐10. Additionally, mRNA levels of the pro‐inflammatory cytokines TNF‐α, IL‐1β, and IL‐6 were elevated in colonic tissues and correlated with increased scores of the disease, indicating intense tissue inflammation in colitis. Some studies [24–26] have reported abnormal expression of numerous cytokines in the intestinal tissues of individuals diagnosed with CD and have found that the interaction between cytokines plays a crucial role, potentially leading to intestinal tissue inflammation as a persistent stimulatory signal, triggering an imbalance in the process of intestinal mucosal injury and repair. IBD can trigger the active release of calprotectin, which is crucial for immune modulation by promoting leukocyte recruitment [27]. Additionally, extracellular calprotectin can interact with TLR4, RAGE, surface heparan sulfate proteoglycan, and carboxylated N‐glycans on endothelial cells, thereby triggering the activation of the downstream nuclear factor‐κB (NF‐κB) pathway and the subsequent synthesis of NF‐κB–regulated pro‐inflammatory mediators, including cytokines and chemokines [28].

Chemokines constitute a superfamily of factors with homologous structures and functions among similar cells. Based on the positions of the first two cysteine residues (C) in their primary protein structures, chemokines can be categorized into the following four types: C, CC, CXC, and CX_3_C [29]. In this study, three highly interacting hub genes belonging to the chemokine family (*CXCL1*, *CXCL2*, and *CXCR2*) were identified, and synergy among these genes represents a key pathway regulating the inflammatory response and the pathological progression of CD. CXCL1 is strongly expressed in macrophages, neutrophils, and epithelial cells, whereby it mediates the inflammatory cascade through neutrophil chemotactic activity. Its expression increases significantly during an inflammatory response, promoting the inflammatory process and inducing leukocyte (especially neutrophil) migration to the site of infection or injury, and amplifying the inflammatory response by the activation of local immune responses, thereby playing a pivotal role in the immune response and modulation of inflammation [30]. Boucher et al. [31] found a highly significant increase in CXCL1 levels in serum samples from patients with CD, as compared to the serum of healthy individuals serving as controls. Further evidence of the dual role of CXCL1 in CD pathogenesis is promoting neovascularization in microvascular endothelial cells, indicating that it not only regulates the recruitment of inflammatory cells and the release of inflammatory mediators but also disrupts the balance of intestinal tissue blood supply, and stability of the internal environment, ultimately leading to persistent damage and fibrosis of the intestinal mucosa [32]. This mechanism is consistent with the pathological cycle of “inflammation–angiogenesis–tissue damage” characteristic of CD, indicating that CXCL1 is a key molecular link between local inflammation and destruction of the intestinal structure. CXCL1 and CXCL2 belong to the CXC chemokine family and share 90% similarity in their amino acid sequences. Both exhibit chemotactic activity, functioning by binding to the G protein–coupled receptor CXCR2. Li et al. [33] found that CXCL2 expression was significantly upregulated in the colonic tissues of IBD mouse models after sensitization, and western blotting and immunohistochemical staining confirmed its specific association with the inflamed colonic mucosal tissues in the IBD mice, suggesting that CXCL2 may act as an “amplifier” of local intestinal inflammation in CD. Taken together with the CXCL1/CXCL2/CXCR2 regulatory axis identified in this study, we can hypothesize that, in the CD intestinal microenvironment, the coordinated high expression of CXCL1 and CXCL2 forms a “chemokine receptor” signaling complex by interacting jointly with CXCR2, thereby enhancing the recruitment of neutrophils and exacerbating inflammatory infiltration and damage to the mucosal barrier. This synergistic effect may explain the refractory inflammation characteristic of CD. As the core receptor of this regulatory axis, the biological significance of CXCR2 is not limited to local intestinal inflammation, as its extensive expression on the surface of inflammatory cells, synovial fibroblasts [34], and cancerous cells of the lung, colon, and ovary [35–38], suggests that it may be involved in regulating the extraintestinal complications of CD. Dong et al. [39] confirmed that IL‐1β can activate neutrophil recruitment by upregulating the expression of CXCL2 (a ligand for CXCR2). Yao et al. [40] found that *CXCR2* is an important hub gene in arteriosclerosis obliterans of the lower extremities complicated with CD and is positively correlated with the degree of neutrophil infiltration. Taken together, the results of this study lead to the proposal of a key hypothesis in which CXCR2 participates not only in the regulation of intestinal inflammation in CD by mediating the chemotactic signals of CXCL1/CXCL2 but may also be a potential molecular target for the extraintestinal complications of CD (such as vascular lesions and joint inflammation) by regulating systemic inflammatory responses and vascular abnormalities, thereby suggesting directions for the multisystemic treatment of CD.

The miRNA–hub gene regulatory network identified two potentially key regulatory miRNAs, hsa‐miR‐1‐3p and hsa‐miR‐335‐5p. Their biological significance and association with CD pathology can be explained as follows: As precise regulators of gene expression, miRNAs inhibit the expression of their target genes by binding to the 3′ untranslated regions of mRNAs, playing key roles in the maintenance of the intestinal barrier function and the regulation of inflammatory balance [41–43]. While there is currently no direct evidence linking has‐miR‐1‐3p to CD, in vitro experiments by Chen et al. [44] discovered that Omega‐3 fatty acids can block the Smad pathway, which is associated with apoptosis and inflammatory responses, through downregulating has‐miR‐1‐3p expression, reducing the levels of pro‐inflammatory cytokines, such as TNF‐α, IL‐1β, and IL‐6, ultimately mitigating sepsis‐induced inflammation and oxidative stress damage in intestinal epithelial cells. This finding is relevant to the core pathological features of CD, specifically, damage to the intestinal epithelial barrier and upregulation of pro‐inflammatory cytokines, suggesting that hsa‐miR‐1‐3p may be involved in the pathogenesis of CD by regulating intestinal epithelial barrier function and inflammatory responses. Abnormal expression of hsa‐miR‐1‐3p in the intestinal microenvironment of CD may promote the activity of the Smad pathway and exacerbate intestinal epithelial cell damage and the release of inflammatory factors. Therefore, its expression level could potentially be used as a biomarker for assessing the extent of intestinal barrier impairment in CD, and it may also be a target for nutritional interventions such as Omega‐3 fatty acids.

The biological significance of hsa‐miR‐335‐5p focuses on the early warning of potential carcinogenesis associated with CD. Sun et al. [45] found that exosome‐transmitted miR‐335‐5p originated from metastatic colorectal cancer cells, promoting the epithelial–mesenchymal transition (EMT) by targeting and modulating the RAS p21 protein activator, while Gao et al. [46] have discovered that miR‐335‐5p levels are reduced in gastric cancer tissues and cell lines and are closely associated with lymph node metastasis, tumor invasion depth, and poor prognosis. Its target gene *MAPK10* (a cell cycle‐related gene) is inversely correlated with miR‐335‐5p expression. Combined with the clinical feature that patients with CD have a significantly increased risk of colorectal cancer, an innovative hypothesis can be proposed: The chronic inflammatory microenvironment of CD may inhibit hsa‐miR‐335‐5p expression, blocking the inhibitory actions on cancer‐related target genes such as *MAPK10*, and thereby promoting the EMT and abnormal proliferation of intestinal mucosal cells and increasing the risk of carcinogenesis.

## 5. Limitations and Future Directions

However, this study has several limitations. First, among the three included datasets, GSE179285 had an unbalanced distribution of samples, with 33 samples in the CD group and only eight in the control group. The relatively small overall sample size may have introduced potential heterogeneity within the dataset, thereby necessitating further validation in independent clinical cohorts. Thus, the present findings need to be confirmed in large‐scale, well‐balanced independent clinical cohorts to enhance the generalizability of the results. Second, while validation datasets were utilized to assess the predictive value of the identified markers, the functional relevance of these key biomarkers and their underlying molecular mechanisms (e.g., the regulatory cascade of the CXCL1/CXCL2/CXCR2 axis and the targeted regulatory relationships between hsa‐miR‐1‐3p/hsa‐miR‐335‐5p and the hub genes) requires experimental verification. In subsequent studies, we aim to conduct both in vitro and in vivo functional experiments (e.g., cell transfection, gene knockout, and CD mouse model verification) to clarify the roles of these candidate molecules in regulating intestinal inflammation, epithelial barrier function, and the inflammation–carcinogenesis transition in CD, exploring the potential for the clinical translation of these molecules and ultimately develop them into reliable diagnostic biomarkers or therapeutic targets for CD.

## 6. Conclusions

This study used the integrated analysis of multiple datasets to identify 60 DEGs, three hub genes (CXCL1, CXCL2, CXCR2), and two miRNAs (hsa‐mir‐1‐3p and hsa‐mir‐335‐5p) potentially contributing to the progression or onset of CD. Functional enrichment analyses revealed that these hub genes primarily mediate the progression of intestinal inflammation in CD through the cytokine–cytokine receptor interactions and chemokine signaling pathways, forming a synergistic regulatory axis (CXCL1/CXCL2/CXCR2) that modulates neutrophil recruitment and amplification of the inflammatory cascade. The identified miRNAs are potentially involved in the regulation of the intestinal epithelial barrier function and CD‐related carcinogenesis risk. Collectively, these findings deepen our understanding of the molecular mechanisms underlying CD pathogenesis and also suggest promising candidate biomarkers for the early diagnosis, disease activity assessment, and prognosis prediction of CD.

## Author Contributions

H.W. and M.S. collaborated in the conception and design of the study, executed the research, conducted the statistical analysis, and collaborated on the initial draft of the manuscript.

## Funding

No funding was received for this research.

## Disclosure

All contributing authors reviewed and endorsed the final version of the manuscript.

## Ethics Statement

No ethical approval or informed consent statement was required for this article.

## Conflicts of Interest

The authors declare no conflicts of interest.

## Supporting Information

Supporting data 1: including the complete ROC outputs.

## Supporting information


**Supporting Information** Additional supporting information can be found online in the Supporting Information section.

## Data Availability

These data were derived from the following resources available in the public domain: Gene Expression Omnibus (GEO) database, http://www.ncbi.nlm.nih.gov/geo/.
