# Supplementary material for: Candidate Biomarkers for Crohn’s Disease: Hub Genes and Regulatory miRNAs Identified by Bioinformatics Analysis
Source: Biochem Res Int. 2026 Feb 5;2026:4628067. doi: 10.1155/bri/4628067 (PMC12874192; doi:10.1155/bri/4628067)
Supplement: Supplementary file 1 — Supporting Information Additional supporting information can be found online in the Supporting Information section. [file BRI-2026-4628067-s001.pdf]

Supplementary Materials – ROC analysis

GSE186582

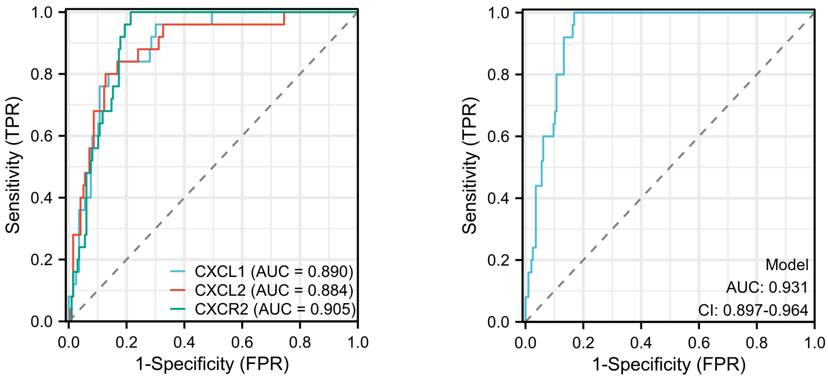

Statistical Description

group	variable	n	min	max	median	iqr	q1	q3	mean	sd	se
CD	CXCL1	196	5.173888	13.46843	9.525277	2.18779425	8.43323825	10.6210325	9.5033359	1.61324091	0.11523149
CD	CXCL2	196	4.165888	12.45995	8.1247065	2.2009745	7.003367	9.2043415	8.16242247	1.60627919	0.11473423
CD	CXCR2	196	3.298197	11.57479	5.884192	2.51543825	4.8547255	7.37016375	6.26313287	1.81831257	0.12987947
Control	CXCL1	25	4.628784	9.498368	7.124972	0.843872	6.508646	7.352518	7.06369412	1.12828434	0.22565687
Control	CXCL2	25	4.224501	9.198606	5.612804	1.033309	5.117331	6.15064	5.82148232	1.06811763	0.21362353
Control	CXCR2	25	3.658338	4.694815	4.079168	0.463778	3.954853	4.418631	4.12396976	0.29802774	0.05960555

Performance measures (95% CI) of the ROC-based optimal cutpoints

variable	cut-off	sensitivity	specificity	PPV	NPV	Youden's index	AUC	95% CI
CXCL1	8.0313	0.84	0.83163	0.38889	0.97605	0.67163	0.890	0.836-0.944
CXCL2	6.2314	0.8	0.87245	0.44444	0.97159	0.67245	0.884	0.817-0.952
CXCR2	4.7076	1	0.78571	0.37313	1	0.78571	0.905	0.865-0.945
Model	-2.1685	1	0.83163	0.43103	1	0.83163	0.931	0.897-0.964

AUC = Area under the ROC curve, PPV = positive predictive value, NPV = negative predictive value.

Multivariate logistic analysis

Characteristics	Beta	OR Multivariate analysis	CI Low Multivariate analysis	CI High Multivariate analysis	P value Multivariate analysis
CXCL1	-0.623250982862331	0.536198429106784	0.264179535414429	1.08830820269843	0.0844092908783934
CXCL2	-0.108207244285462	0.89744158751591	0.437304764973905	1.84173937151391	0.767992194373881
CXCR2	-1.78793893753276	0.167304639871629	0.0512430466757913	0.546236891410268	0.00306025790882305

GSE179285

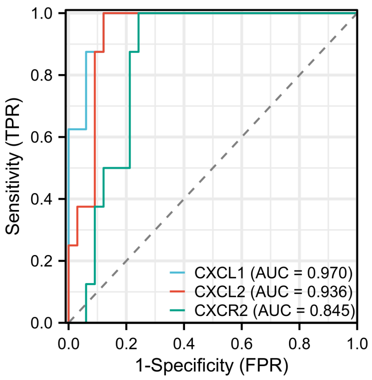

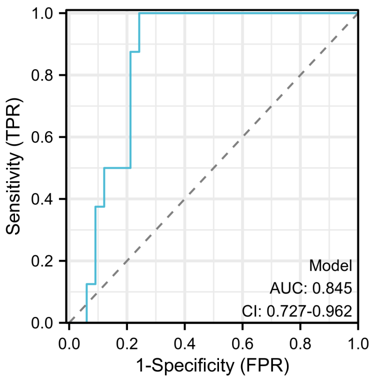

Statistical Description

group	variable	n	min	max	median	iqr	q1	q3	mean	sd	se
CD	CXCL1	33	0.88991617	59.7745297	8.09634089	11.9472379	5.15535137	17.1025893	13.1341134	12.7280969	2.21567728
CD	CXCL2	33	0.81076132	32.1599823	5.15265318	7.5941229	2.91620007	10.510323	7.96484848	7.76867982	1.35235357
CD	CXCR2	33	0.84838653	1.54919826	1.07982122	0.22229787	1.02796017	1.25025804	1.1293608	0.1685487	0.02934056
Control	CXCL1	8	0.31296502	2.19742554	0.48461603	0.5188571	0.42487228	0.94372938	0.77510019	0.6247491	0.22088216
Control	CXCL2	8	0.54628947	1.45158681	1.00902617	0.54560901	0.77719303	1.32280204	1.02060159	0.34800529	0.12303845
Control	CXCR2	8	0.89566457	0.99805813	0.96630819	0.04132196	0.94574443	0.9870664	0.96174583	0.03475847	0.01228898

Performance measures (95% CI) of the ROC-based optimal cutpoints

variable	cut-off	sensitivity	specificity	PPV	NPV	Youden's index	AUC	95% CI
CXCL1	2.3157	1	0.87879	0.66667	1	0.87879	0.970	0.925-1.000
CXCL2	1.4863	1	0.87879	0.66667	1	0.87879	0.936	0.861-1.000
CXCR2	1.013	1	0.75758	0.5	1	0.75758	0.845	0.727-0.962
Model	-1.29	1	0.75758	0.5	1	0.75758	0.845	0.727-0.962

AUC = Area under the ROC curve, PPV = positive predictive value, NPV = negative predictive value.

Multivariate logistic analysis

Characteristics	Beta	OR Multivariate analysis	CI Low Multivariate analysis	CI High Multivariate analysis	P value Multivariate analysis
CXCR2	-14.2445379952376	6.51142063373143e-07	3.03795957793572e-12	0.13956274789605	0.0229427015679996

GSE112366

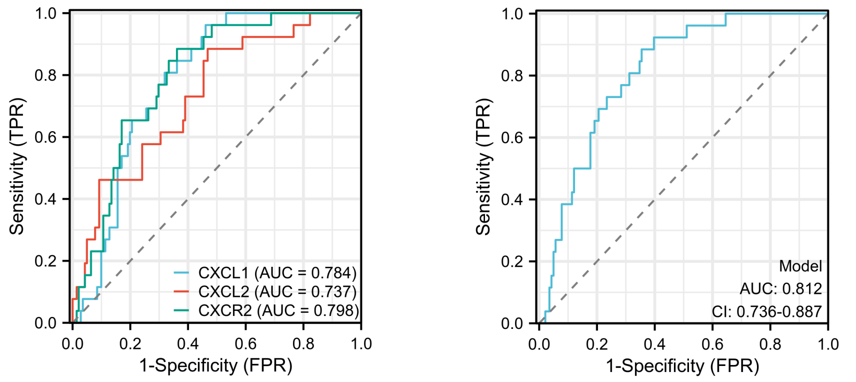

Statistical Description

group	variable	n	min	max	median	iqr	q1	q3	mean	sd	se
CD	CXCL1	141	4.5679	11.7471	8.1355	2.8869	6.5021	9.389	7.99101631	1.7975462	0.1513807
CD	CXCL2	141	3.0224	7.4452	3.8313	1.1194	3.4601	4.5795	4.19752908	1.00990932	0.08504971
CD	CXCR2	141	4.7121	11.3398	6.3145	2.8547	5.5515	8.4062	6.93619716	1.66324109	0.14007017
Control	CXCL1	26	4.6586	8.4625	5.93285	1.202375	5.577725	6.7801	6.23764231	0.94398438	0.18513057
Control	CXCL2	26	2.8817	5.1226	3.435	0.51345	3.1873	3.70075	3.51955769	0.50714215	0.09945876
Control	CXCR2	26	4.8473	7.8005	5.33795	0.417825	5.230975	5.6488	5.48760769	0.57807192	0.11336923

Performance measures (95% CI) of the ROC-based optimal cutpoints

variable	cut-off	sensitivity	specificity	PPV	NPV	Youden's index	AUC	95% CI
CXCL1	7.8665	0.96154	0.53901	0.27778	0.98701	0.50055	0.784	0.711-0.858
CXCL2	3.8014	0.88462	0.53191	0.25843	0.96154	0.41653	0.737	0.636-0.838
CXCR2	5.7772	0.88462	0.6383	0.31081	0.96774	0.52291	0.798	0.720-0.876
Model	-1.5152	0.88462	0.64539	0.31507	0.96809	0.53001	0.812	0.736-0.887

AUC = Area under the ROC curve, PPV = positive predictive value, NPV = negative predictive value.

Multivariate logistic analysis

Characteristics	Beta	OR Multivariate analysis	CI Low Multivariate analysis	CI High Multivariate analysis	P value Multivariate analysis
CXCL1	-0.258967169172592	0.771848363048127	0.462983142131274	1.28676368819311	0.320665580287797
CXCL2	-0.249416533148229	0.779255320103211	0.270944788178113	2.24119038418253	0.643552148606228
CXCR2	-0.92932078907762	0.394821786590993	0.1504511042935	1.03611232299635	0.05903989799442
